# The role of preoperative pulmonary function tests in the surgical treatment of extremely severe scoliosis

**DOI:** 10.1186/1749-799X-8-32

**Published:** 2013-09-05

**Authors:** Lifeng Lao, Xisheng Weng, Guixing Qiu, Jianxiong Shen

**Affiliations:** 1 Department of Orthopedic Surgery, Renji Hospital, Shanghai Jiao Tong University School of Medicine, Shanghai, China; 2 Department of Orthopedic Surgery, Peking Union Medical College Hospital, Chinese Academy of Medical Sciences, Beijing 100730, China

**Keywords:** Severe scoliosis, Pulmonary function, Pulmonary function tests, Pulmonary complication

## Abstract

**Background:**

The patients with extremely severe spinal deformity are commonly considered high-risk candidates for surgical treatment because of their underlying lung disease. Currently, little has been reported about the postoperative pulmonary complication events in this population. This retrospective study sought to evaluate preoperative pulmonary function tests in the surgical treatment of extremely severe scoliosis.

**Methods:**

Preoperative forced vital capacity (FVC), FVC ratio, forced expiratory volume at the end of the first second (FEV1), FEV1 ratio, peak expiratory flow (PEF), and PEF ratio were performed and evaluated on 60 patients with extremely severe scoliosis (coronary main Cobb angle ≥100°).

**Results:**

Among the 60 patients, 11 (18.3%), 13 (21.7%), and 22 (36.7%) had severe, moderate, and mild pulmonary dysfunction, respectively. Compared with the moderate and mild scoliosis groups, significant differences were observed in Cobb, FVC, FVC ratio, FEV1, FEV1 ratio, and PEF ratio in the extremely severe scoliosis group. Various postoperative pulmonary complications occurred in nine cases (15%). Patients with severe or moderate dysfunction as measured by the FVC ratio had a higher incidence of postoperative pulmonary complications. A transthoracic procedure was not related to postoperative pulmonary complications, but thoracoplasty significantly increased the incidence of postoperative pulmonary complications (*P* < 0.001, OR = 20, 95% CI = 3.45–115.97).

**Discussion:**

Pulmonary function was impaired in extremely severe scoliosis. Patients with severe restrictive pulmonary dysfunction had a higher incidence of postoperative pulmonary complications. Thoracoplasty was an important risk factor in the prediction of postoperative pulmonary complications.

## Introduction

Great progress has been made in the treatment of scoliosis, but some scoliotic patients, especially those with severe scoliosis, develop postoperative pulmonary complications as a result of various factors during hospitalization. Despite the need to treat the extremely severe curve in the spines of these patients, they are commonly considered high-risk candidates for surgical treatment because of their underlying lung disease [1]. Currently, little has been reported about the postoperative pulmonary complication events in this population. This non-randomized, retrospective study was undertaken to evaluate the role of preoperative pulmonary function tests in severe scoliotic patients.

## Materials and methods

### Subjects

We reviewed the records of 940 scoliotic patients who underwent surgical treatment from 2002 to 2012. Among these patients, 60 (18 males and 42 females) patients with extremely severe scoliosis were identified. The average coronal Cobb angle was 110.9° (range 100° to 150°). The diagnoses for these 60 patients included idiopathic scoliosis in 29 cases, congenital scoliosis in 8 cases, neurofibromatosis in 7 cases, adult scoliosis in 6 cases, scoliosis with neurofibromata in 5 cases, scoliosis with revision operations in 3 cases, and Marfan syndrome in 2 cases. The mean age of the patients was 17.3 years (range 9 to 40 years). Each patient's hospital and clinical chart was reviewed, and information on indications for surgery, age, preoperative medical conditions, perioperative records, and laboratory results was collected. This study was approved by the ethics committee of the hospital. Written informed study consents to participate in this study were obtained from the patients or the child guardians.

### Pulmonary function tests

All of the patients received pulmonary function tests (PFTs) before surgery. The PFTs included the following six parameters: forced vital capacity (FVC), FVC ratio, forced expiratory volume at the end of the first second (FEV1), FEV1 ratio, peak expiratory flow (PEF), and PEF ratio. The 60 patients were divided into two subgroups according to the severity of the restrictions of their preoperative pulmonary function. The severe restrictive group fits the following criteria: FVC ratio ≤ 65%, FEV1 ratio ≤ 65%, and PEF ratio ≤ 65%. The moderate restrictive group followed the subsequent criteria: FVC ratio > 65%, FEV1 ratio > 65%, and PEF ratio > 65%. The surgical procedures included anterior spinal fusion in 3 cases, posterior spinal fusion in 27 cases, one-stage anterior and posterior spinal fusion in 9 cases, and two-stage anterior and posterior spinal fusion in 21 cases. The chosen surgical approach depended on the curve type and flexibility. More specifically, anterior spinal fusion was indicated for single flexible thoracic or thoracolumbar curves, and combined anterior/posterior spinal fusion was administered for rigid curves. The staging of the procedures depended on the complexity of the procedures, the intraoperative status of the patients, and the anticipated interval traction. The second of the staged procedures was performed about 1 week after the first. The mean operation time was 396 min, and the mean blood loss was 989 mL. All patients underwent intraoperative neurologic monitoring such as SSEP and MEP. Tracheal intubation was kept into intensive care unit and pulled out by an anesthetist.

The following postoperative pulmonary complications were identified: the presence of pleural effusion, atelectasis, hemothorax, pneumothorax, pneumonia, hypoxemia (80% < SO_2_ ≤ 90% for over 8 h), and respiratory failure (an increased requirement of postoperative ventilatory support with intubation). The presence of any preoperative and/or postoperative pulmonary symptoms and signs (dyspnea, breathlessness on exertion, crepitations, or rhonchi), and increased requirement of postoperative ventilatory support were noted by checking the ward physicians' notes. As for the respiratory failure, the inclusion criteria were PaO_2_ lower than 60 mmHg (8.0 kPa) and PaCO_2_ normal or lower than 50 mmHg (6.7 kPa). They were treated with ventilatory support, no matter how many days it lasted. Postoperative chest radiographs were examined to note the presence of pleural effusion, atelectasis, hemothorax, pneumothorax, and/or pneumonia. Postoperative chest radiographs were obtained for patients who had suspicious chest auscultation findings and for all patients who underwent thoracoplasty.

### Statistical analysis

Statistical analysis was performed to determine the correlations between scoliotic severity and preoperative pulmonary function, between preoperative abnormal pulmonary function and postoperative pulmonary complications, and between operative approaches and postoperative pulmonary complications. The data were analyzed using the SPSS statistical package 13.0. All tests were double sided and considered statistically significant at *P* < 0.05. If the theoretical number was less than 5, the chi-square test was corrected accordingly.

## Results

A comparison between the extremely severe scoliosis group and the mild and moderate scoliosis groups in terms of preoperative pulmonary function tests showed that significant differences were observed in the Cobb angle, FVC, FVC ratio, FEV1, FEV1 ratio, and PEF ratio (*P* < 0.01). In contrast, no significant differences were observed in the PEF (*P* > 0.05) (Table 1).

**Table 1:** Comparison of preoperative pulmonary function tests for scoliosis

	**Cobb**	**FVC**	**FVC ratio**	**FEV1**	**FEV1 ratio**	**PEF**	**PEF ratio**
	**(deg)**	**(L)**	**(%)**	**(L)**	**(%)**	**(L/min)**	**(%)**
Extremely severe	110.917	2.l90	73.149	1.914	73.667	4.699	81.303
Mild and moderate	55.336	2.761	83.471	2.440	87.593	6.592	88.608
*t*	26.227	−3.817	−4.136	−5.742	−5.476	−0.538	−2.667
*P*	0.000	0.000	0.000	0.000	0.000	0.591	0.008

Various pulmonary complications were observed in nine of the extremely severe scoliosis cases (15%), including two cases of pleural effusion (3.3%), two cases of respiratory failure with ventilatory support (3.3%), one case of atelectasis (1.7%), one case of hemothorax (1.7%), one case of pneumothorax (1.7%), one case of hypoxemia (1.7%), and one case of pneumonia (1.7%) (Table 2).

**Table 2:** Incidence of postoperative pulmonary complications in extremely severe scoliosis

**Pulmonary complication**	**Number of patients**
Pleural effusion	2
Respiratory failure	2
Atelectasis	1
Hemothorax	1
Pneumothorax	1
Hypoxemia	1
Pneumonia	1
Total	9

In the severe restrictive group according to the FVC ratio, more significant postoperative pulmonary complications were observed (*P* < 0.05). In the severe restrictive group according to the FEV1 ratio and the PEF ratio, no significant postoperative pulmonary complications were observed (*P* > 0.05) (Table 3).

**Table 3:** Correlation between restrictive preoperative PFTs and postoperative pulmonary complications in patients with extremely severe scoliosis

**Preoperative PFTs**	**Pulmonary complication**		***χ***^**2**^	***P***
	**Yes**	**No**		
FVC ratio				
≤65%	7	17	4.581	0.032
>65%	2	34		
FEV1 ratio				
≤65%	6	15	3.173	0.075
>65%	3	36		
PEF ratio				
≤65%	5	10	3.529	0.060
>65%	4	41		

No significant difference was observed in terms of postoperative pulmonary complications between patients receiving a transthoracic procedure and those receiving a non-transthoracic procedure (*χ*^2^ = 0.160, *P* = 0.689, OR = 1.78, 95% CI = 0.40–7.90). However, a significant difference was observed in terms of postoperative pulmonary complications between those who underwent thoracoplasty and those who did not (*χ*^2^ = 12.319, *P* < 0.001, OR = 20, 95% CI = 3.45–115.97). Nevertheless, no significant differences were observed between the thoracoplasty and non-thoracoplasty groups in terms of age, preoperative coronal Cobb angle, and preoperative pulmonary function. This indicates that thoracoplasty significantly increases the incidence of postoperative pulmonary complications (Tables 4 and 5).

**Table 4:** Correlation between the transthoracic procedure and postoperative pulmonary complications

	**Pulmonary complication**		**Total**
	**Yes**	**No**	
Transthoracic	6	27	33
Non-transthoracic	3	24	27
Total	9	51	60

*χ*^2^ = 0.160, *P* = 0.689, OR = 1.78, 95% CI = 0.40–7.90.

**Table 5:** Correlation between thoracoplasty and postoperative pulmonary complications

	**Pulmonary complication**		**Total**
	**Yes**	**No**	
Thoracoplasty	5	3	8
Non-thoracoplasty	4	48	52
Total	9	51	60

*χ*^2^ = 12.319, *P* < 0.001, OR = 20, 95% CI = 3.45–115.97.

## Discussion

In patients with severe scoliosis, the preoperative pulmonary function is remarkably affected [2]. Our study found that in the group of patients with extremely severe scoliosis, the FVC, FEV1, and their ratios are significantly affected. PFTs can be used to predict the incidence of postoperative pulmonary complications. Stein et al. [3] studied two groups of patients who underwent operations and found that the patients with abnormal PFTs had a higher incidence of pulmonary complications. Padman and McNamara [4] also reported a significant correlation between abnormal preoperative PFTs and postoperative pulmonary complications in neuromuscular scoliotic patients with posterior fusion. Vedantam and Crawford [5] found that these two factors were significantly correlated; however, in their study, the average Cobb angle was only 48°, and most of the patients demonstrated normal preoperative pulmonary function. Our study of patients with extremely severe scoliosis showed that the postoperative pulmonary complication rate increased with the increasing severity of preoperative restrictive pulmonary function. Moreover, as the FVC ratio worsens, the incidence of postoperative pulmonary complications increases. Within the PFT, the FVC ratio indicates the level of restriction for the pulmonary function. Thus, the presence of preoperative restrictive pulmonary function could be a useful predictor of postoperative pulmonary complications. Postoperative pulmonary complications are thought to be associated with the surgical approach, preoperative PFTs, and thoracoplasty [1]. In this study, various pulmonary complications occurred in nine patients with extremely severe scoliosis, which results in a 15% incidence rate. Zhang et al. [6] reported a postoperative pulmonary complication incidence of 6.4% in 298 patients with scoliosis, but the average Cobb angle for this sample population was only 73.3°.

The type of surgical procedure used as treatment has a significant influence on the postoperative pulmonary complication rate. Newton et al. [2] reported that for patients undergoing open thoracotomy with thoracoplasty, approximately 54% had a 15% or more decrease in predicted PFT. Mohamad et al. [7] found that the incidence of postoperative pulmonary complications in patients who underwent single-stage procedures was higher than in the patients who underwent staged procedures. Zhang et al. [6] also reported that the incidence of postoperative pulmonary complications in patients treated with a transthoracic procedure was 18 times higher than that in patients treated with the posterior approach. Therefore, in patients with moderate or severe restrictive preoperative pulmonary function, a posterior-only approach or a two-stage anterior and posterior spinal fusion may be the most appropriate. Rawlins et al. [8] noted that patients going through transthoracic or combined thoracoabdominal procedures were more likely to have postoperative pulmonary complications than those undergoing a posterior approach. Our data showed that the transthoracic procedure did not affect the pulmonary complication rate, but the incidence of postoperative pulmonary complications associated with thoracoplasty was 20 times higher than that of the non-thoracoplasty approach. Therefore, the operative procedure chosen for the treatment plan plays a very important role in predicting postoperative pulmonary complications, and the thoracoplasty procedure is the only risk factor for postoperative pulmonary complications. This result may be due to the limited number of patients in each subgroup and the two-staged surgical process with the initial stage as the anterior release.

Thoracoplasty has been the traditionally chosen treatment for rib hump. Vedantam and Crawford [5] reported that the performance of a thoracoplasty was the only risk factor for postoperative pulmonary complications in patients undergoing posterior spinal fusion. However, Hod-Feins et al. [9] reported that thoracoplasty did not correlate with the higher rate of postoperative pulmonary complications and suggested that thoracoplasty could be administered whenever indicated. Suk et al. [10] found that thoracoplasty could result in better clinical outcomes without pulmonary function compromise in the treatment of adolescent thoracic idiopathic scoliosis. Our study found that patients who underwent thoracoplasty were 20 times more likely to have postoperative pulmonary complications than those who have not undergone thoracoplasty (95% confidence interval 3.45–115.97; *P* < 0.001). This difference may be caused by the fact that in our study, the average Cobb angle was 110.9° in these cases of extremely severe scoliosis. Thus, in our view, patients with severe scoliosis, especially those who plan to undergo thoracoplasty, should be counseled preoperatively about the increased risk for postoperative pulmonary complications.

To our knowledge, surgical treatment of severe and rigid scoliosis had various high risks [11], and this is the first clinical report on postoperative pulmonary complications in extremely severe scoliosis surgery and alerts anesthesiologists and surgeons to the need to clinically manage this potential problem. Overall, the pulmonary complications were controllable, and no deaths or lingering side effects were observed. Therefore, we should not overestimate the risks associated with these procedures and deny treatment for patients with extremely severe scoliosis and poor pulmonary function, for whom the surgical intervention is most needed. However, successful treatment for these patients requires teamwork from skilled spinal surgeons and anesthesiologists. Recently, there are more studies about lung function affected by scoliosis treatment [12-15]. This study is a retrospective research, and the results are limited by the relatively small sample size and the non-randomized design. Other limitations included lack of postoperative pulmonary function test and relatively short-term follow-up. A prospective, randomized, and multicenter study would be expected in the future.

## Conclusions

Pulmonary function was impaired in patients with extremely severe scoliosis. Patients with severe restrictive pulmonary dysfunction had higher incidences of postoperative pulmonary complications. Thoracoplasty was an important risk factor in the prediction of postoperative pulmonary complications. This information should be considered when surgeons are discussing the risks of postoperative pulmonary complications associated with scoliotic surgery for patients with extremely severe scoliosis.

## Competing interests

The authors declare that they have no competing interests.

## Authors’ contributions

The design of the study and preparation of the manuscript were done by LL and XW. LL and GQ assisted in the study processes, data collection, and preparations. LL and JS assisted in the manuscript preparation. All authors read and approved the final manuscript.
